# Associations of age, sex, sexual abuse, and genotype with monoamine oxidase a gene methylation

**DOI:** 10.1007/s00702-021-02403-2

**Published:** 2021-08-23

**Authors:** David Checknita, Jari Tiihonen, Sheilagh Hodgins, Kent W. Nilsson

**Affiliations:** 1 Department of Neuroscience, Uppsala University, Uppsala, Sweden; 2 Department of Clinical Neuroscience, Karolinska Institutet, Psychiatry Building R5:00 c/o Jari Tiihonen, Karolinska Universitetssjukhuset, 171 76 Stockholm, Sweden; 3 Centre for Clinical Research, Västmanland County Council, Uppsala University, Uppsala, Sweden; 4 Center for Psychiatry Research, Stockholm City Council, Stockholm, Sweden; 5 Department of Forensic Psychiatry, Niuvanniemi Hospital, University of Eastern Finland, Kuopio, Finland; 6 Département de Psychiatrie et Addictologie, Centre de Recherche de l’Institut Universitaire en Santé Mentale de Montréal, Université de Montréal, Montréal, QC Canada

**Keywords:** Epigenetics, Methylation, Age, Sex differences, Child abuse, Gene–environment interaction

## Abstract

Epigenome-wide studies report higher methylation among women than men with decreasing levels with age. Little is known about associations of sex and age with methylation of monoamine oxidase A (*MAOA*). Methylation of the first exonic and partial first intronic region of *MAOA* has been shown to strengthen associations of interactions of MAOA-uVNTR genotypes and adversity with aggression and substance misuse. Our study examined associations of sex and age with *MAOA* first exon and intron methylation levels in 252 women and 157 men aged 14–73 years. Participants included adolescents recruited at a substance misuse clinic, their siblings and parents, and healthy women. Women showed ~ 50% higher levels of exonic, and ~ 15% higher intronic, methylation than men. Methylation levels were similar between younger (M = 22.7 years) and older (M = 46.1 years) participants, and stable across age. Age modified few associations of methylation levels with sex. *MAOA* genotypes modified few associations of methylation with sex and age. Higher methylation levels among women were not explained by genotype, nor interaction of genotype and sexual abuse. Findings were similar after adjusting for lifetime diagnoses of substance dependence (women = 24.3%; men = 34.2%). Methylation levels were higher among women who experienced sexual abuse than women who did not. Results extend on prior studies by showing that women display higher levels of methylation than men within first intronic/exonic regions of *MAOA,* which did not decrease with age in either sex. Findings were not conditioned by genotype nor interactions of genotype and trauma, and indicate X-chromosome inactivation.

**Supplementary Information:**

The online version contains supplementary material available at 10.1007/s00702-021-02403-2.

## Introduction

Methylation of DNA at cytosine–guanine dinucleotides (CpGs) across the genome is a dynamic biological process influenced by environmental and biological factors, that in turn impacts gene activity and the subsequent regulation of biological systems (Xiao et al. 2018; Pagiatakis et al. 2019). Together, these factors contribute to the high degree of inter-individual variation in CpG methylation across the genome associated with mental and physical disorders (Han et al. 2019; Pagiatakis et al. 2019). However, recent reviews of studies of methylation levels characterizing individuals with various disorders report inconsistent findings. These inconsistencies may result from inadequate control for, or examination of sex- and age-related variations in methylation (Barnett Burns et al. 2018; Brown et al. 2019; Fransquet et al. 2019; Gegenhuber and Tollkuhn 2019; Han et al. 2019). Furthermore, little is known about the associations of sex and age with genes on the X-chromosome, for example monoamine oxidase A (*MAOA*), that has been excluded from some epigenome-wide methylation studies (Chen et al. 2020), even though *MAOA* plays a major role early in life by setting life-long levels of brain monoamines (Meyer-Lindenberg et al. 2006; Oreland et al. 2007; Booij et al. 2015). Extant evidence also implicates gene-by-environment interactions of a functional variable nucleotide polymorphism in the promoter of *MAOA* (MAOA-uVNTR) and adversity in modifying risk for negative behavioural outcomes, principally antisocial behaviour, and mental disorders such as depression (Byrd and Manuck 2014; Booij et al. 2015; Nilsson et al. 2018). Polymorphisms of *MAOA* have also been implicated in treatment response to mirtazapine among individuals with depression (Tzeng et al. 2009), further highlighting the gene’s clinical importance to understanding etiology and treatment.

## Sex and DNA methylation

Females typically show higher methylation levels than males in genome-wide studies, although the magnitude and direction of sex differences can vary greatly at specific genomic regions (Nugent and McCarthy 2011). Accordingly, sex differences in methylation have been identified in genes contributing to regulation of biological pathways involved in sex differentiation, endocrine function, and neurodevelopment, in genes that regulate epigenetic processes and mechanisms, and in genes regulating aging (Nugent and McCarthy 2011; Yousefi et al. 2015; Kader and Ghai 2016; Van Dongen et al. 2016; Ratnu et al. 2017; Suderman et al. 2017). Only one study has examined sex differences in methylation of *MAOA*: The sample was small, 23 of the 66 participants presented with depression, and women showed higher methylation levels than men within the exonic region (Melas and Forsell 2015). Similar levels of *MAOA* first exon methylation in women, ranging between 40 and 50%, have been reported (Ziegler and Domschke 2018).

Sex differences in methylation have been observed among individuals presenting with mental or physical disorders (Ratnu et al. 2017). Several mental disorders are characterized by alterations of methylation of the *MAOA* gene including depression, post-traumatic stress disorder, substance abuse disorders, antisocial personality disorder, conduct disorder, borderline personality disorder, and schizophrenia (Ziegler and Domschke 2018), and by different functional variable number tandem repeat (uVNTR) variants of *MAOA* in males and females (reviewed in Veroude et al. 2016). As the gene sits on the X-chromosome, sex differences in methylation levels may result from X-chromosome inactivation, an epigenetic process whereby one of the two X-chromosomes carried by females is silenced. Inactivation serves as a dosage compensation mechanism in females, relative to males who carry only one X-chromosome (Cotton et al. 2011). X-inactivation is also linked to the exacerbation of numerous sexually dimorphic phenotypes (Carrel and Willard 2005) ranging from cell structure (Garieri et al. 2018) to disease susceptibility (Schurz et al. 2019) and has been hypothesized to play a role in sexually dimorphic behaviors (de Almeida et al. 2015). Notably, women also show greater variability in X-linked gene expression than men (Peeters et al. 2014). This variability may be partly attributable to the approximately 15% of X-linked genes that partially, or fully, escape inactivation (Carrel and Willard 2005) which also varies greatly by cell type (Garieri et al. 2018). As such, sex differences in methylation, particularly among X-linked genes, are key considerations for furthering understanding of links with environmental events, treatment response, fundamental biological processes, and how the epigenome itself functions (Ratnu et al. 2017).

## Aging and methylation

Sex differences in methylation levels across the genome are observable in new-borns (Yousefi et al. 2015), remain stable through childhood (Suderman et al. 2017), and are observed in adulthood (Ratnu et al. 2017). There is a global reduction of methylation across the genome with increasing age, such that cumulative inter-individual differences are harmonized in a process called epigenetic assimilation, although methylation levels in some regions do not undergo this reduction (Ciccarone et al. 2018). This global reduction of methylation levels over the lifespan is not strongly associated with changes in gene expression (Ciccarone et al. 2018). It has been hypothesized that regulation of gene expression during this period of lowering methylation may be gradually taken up and maintained by other epigenetic processes over time, although these mechanisms are not currently well understood (Ashapkin et al. 2017; Ciccarone et al. 2018; Pagiatakis et al. 2019; Xiao et al. 2019; Chen et al. 2020). Disturbances in the trajectory of epigenetic aging are associated with health risks across the lifespan, and may be influenced by environmental factors encountered as early as pre- and post-natal life (Ciccarone et al. 2018; Chen et al. 2020). Importantly, despite the pattern of genome-wide hypo-methylation with increasing age, age-related fluctuations in DNA methylation may remain more variable and susceptible to environmental influences within particular genomic loci, including regions that span first exonic/intronic boundaries of genes, and are thus regions of particular interest in health-related outcomes (Ashapkin et al. 2017; Ciccarone et al. 2018).

Disturbances to the trajectory of epigenetic aging processes have been implicated in risk for mental and physical illnesses (Miller et al. 2015; Ciccarone et al. 2018; Vaiserman 2018), influenced by environmental factors (Ciccarone et al. 2018; Vaiserman 2018), and may differ by sex (Van Dongen et al. 2016). One study showed that among children in families with low socioeconomic status, those exposed to positive environmental factors showed greater self-control that in turn predicted less aggressive behavior, fewer depressive symptoms, lower rates of substance use, and fewer externalizing problems, increased academic success, and better psychosocial adjustment in young adulthood (Miller et al. 2015). Exposure to positive factors, however, did not protect against accelerated epigenetic aging, nor poor cardio-metabolic health indicators that predict medical illnesses later in life (Miller et al. 2015). The authors of this study suggested a potential “skin deep” resilience, such that observed protective associations with outcomes that begin in late-adolescence and early adulthood mask an increased risk for other negative outcomes that may emerge over protracted periods of time (Miller et al. 2015). The impact of environmental factors present in prenatal and early life on the re-programming of epigenetic aging processes across the lifespan is further supported by twin and non-human animal studies, and is associated with alterations of metabolic functions that underlie a myriad of health conditions that emerge throughout the lifespan (Vaiserman 2018). A recent study of twins found that genetic factors were strongly associated with stability of methylation levels over time, while novel experiences and exposure to environmental factors accounted for fluctuations in methylation levels (Reynolds et al. 2020). In turn, age is increasingly being recognized as an important consideration in epigenetic studies as mental and physical disorders that emerge over time may be associated with aberrant epigenetic aging processes initiated by environmental factors acting in early life (Miller et al. 2015; Ciccarone et al. 2018; Pagiatakis et al. 2019).

## Sex and aging

A recent systematic review and meta-analysis of DNA methylation studies of aging and age-related physical disorders and mortality suggested that the inconsistencies in findings across studies were partly attributable to insufficient statistical control for sex and bias introduced by sampling only males both in human and animal studies (Fransquet et al. 2019). One study found that the interaction of age and sex was associated with methylation levels across the genome, especially in regions associated with key health indicators such as metabolic traits and smoking (Van Dongen et al. 2016). Another recent study found that the majority of sex differences in methylation associated with age were in X-linked genes implicated in sexually dimorphic disorders and traits, including prostate cancer and male pattern baldness (McCartney et al. 2019). Another recent review highlighted that sex differences in age-related methylation may differ widely by genomic region and by tissue type, particularly in the brain (Unnikrishnan et al. 2019). For instance, one study in mice and humans showed that genome-wide hippocampal methylation levels did not differ by sex in early life, but that 95% of age-related changes in methylation levels observed later in life were sexually divergent and particular to specific genomic loci (Masser et al. 2017). Prior methylation studies of persons presenting with mental disorders have often been conducted in tissue samples from post-mortem hippocampal tissues (Barnett Burns et al. 2018). Further understanding of the role of methylation in mental disorders using peripheral tissue samples from afflicted individuals is needed (Szyf 2015; Barnett Burns et al. 2018; Brown et al. 2019). Yet, such studies are presently hindered by the lack of knowledge of sex and age differences across the genome and in candidate genes.

The current understanding of sex, age, and methylation derives primarily from genome-wide studies (Ciccarone et al. 2018; Vaiserman 2018; Chen et al. 2020). Candidate gene studies including regions of interest, such as exonic/intronic junctions, that may be subject to greater fluctuation in methylation across the lifespan have the potential to extend understanding of how environmental and biological factors alter risk for physical and mental health. For example, a recent study found little association of epigenome-wide methylation and aggressive behaviour in general population samples, and none in a sample of adolescents with ADHD nor in a sample of females presenting conduct disorder (van Dongen et al. 2021). By contrast, a recent study from our group found that among men, methylation levels of a region-of-interest (ROI) spanning the *MAOA* first exon were associated with aggressive behaviour (Checknita et al. 2020).

Furthermore, epigenetic studies of candidate genes have the potential to further specify whether associations with sex and age are modified by genotype, and by interactions of genotype and environmental factors as has been reported in epigenome studies (Van Dongen et al. 2016). Surprisingly, one recent study of a large cohort found no direct association of childhood adversity with genome-wide methylation or methylation of six candidate genes (not including *MAOA*). The authors hypothesized that other factors, including genotype, may increase vulnerability of genes to epigenetic changes resulting from maltreatment (Marzi et al. 2018). One recent twin study reported that regions across the genome showing enrichments of DNA methylation associated with environmental factors were also strongly influenced by additive genetic factors (Hannon et al. 2018). Furthermore, genome-wide and gene-specific associations between environmental factors, genotypes, and epigenetic processes have been identified in risk for numerous physical illnesses such as cancer, diabetes, and neurodegenerative disorders (Romanowska and Joshi 2019). A recent systematic review of associations of DNA methylation and brain structure and function across the lifespan among individuals presenting with neurodegenerative or mental disorders noted the importance of controlling for, or examining, the impact of genotype (Wheater et al. 2020).

### Methylation of *MAOA*

Two recent studies from our group found that, among men, methylation levels of a ROI spanning a portion of the *MAOA* core promoter containing the gene’s first exonic and partial first intronic regions further modified the association of the interaction of maltreatment and *MAOA-*uVNTR genotypes with alcohol consumption (Bendre et al. 2018) and with aggressive behavior (Checknita et al. 2020). Similarly, another recent study found that among women who had experienced adversity in childhood genotype-specific methylation profiles of *FKBP5* intron 7 were associated with Post Traumatic Stress Disorder symptom severity (Grasso et al. 2020). Furthermore, recent studies suggest that promoter sequence variants can alter the binding affinity of methyl groups to DNA in upstream regions, which in turn alters the susceptibility of genes to be epigenetically modified by environmental factors, offering one potential mechanism through which genotype and methylation can interact (Ahsan et al. 2017; Ek et al. 2018).

The *MAOA* gene, located on the X-chromosome (Xp11.3), encodes the monoamine oxidase A (MAO-A) enzyme which metabolizes serotonin (and other aminergic transmitters) following reuptake. Aberrant activity of *MAOA* has been posited as a critical factor involved in system-wide aminergic dysregulation that, in turn, contributes to the development of multiple negative outcomes including increased aggressive behavior, substance misuse, and mental disorders such as depression (Meyer-Lindenberg et al. 2006; Byrd and Manuck 2014; Booij et al. 2015; Nilsson et al. 2018; Ziegler and Domschke 2018). The promoter region of *MAOA* contains a functional 30 bp variable tandem number repeats polymorphism (MAOA-uVNTR) that includes short 2–3 repeat low-expressing variants (MAOA-S), and long 3.5–5 repeat high-expressing variants (MAOA-L) (Beach et al. 2010). Direct associations of *MAOA* genotypes with negative outcomes are not well supported. Rather, the interactions of sex-specific genotypes (in males MAOA-S, in females MAOA-L) with exposure to negative environmental factors in childhood are associated with increased risk of negative outcomes such as Antisocial Personality Disorder and antisocial behavior, criminality, aggressive behavior, and substance misuse (Caspi et al. 2002; Sjöberg et al. 2007; Beach et al. 2010; Wakschlag et al. 2011; Åslund et al. 2011; Byrd and Manuck 2014; Tiihonen et al. 2014; Nilsson et al. 2018). The importance of this robust finding is underlined by the large number of individuals carrying these susceptibility alleles. In Caucasian populations, approximately one-third of males (Caspi et al. 2002) and at least two-thirds of females carry *MAOA* susceptibility alleles (Åslund et al. 2011). Recent meta-analyses and reviews note, however, that while associations of negative outcomes with interactions of *MAOA* and childhood trauma are robust, there are notable discrepancies in results. The discrepancies may be accounted for, at least in part, by failure to take account of epigenetic processes that modify gene expression (Nordquist and Oreland 2010; Booij et al. 2015; Ziegler and Domschke 2018).

Altered DNA methylation in an *MAOA* region of interest spanning the first exonic and partial first intronic regions, among men and women, has been associated with mental disorders including anxiety disorders, depression, substance abuse disorders, post-traumatic stress disorder, antisocial personality disorder, borderline personality disorder, and schizophrenia (Melas and Forsell 2015; Checknita et al. 2015, 2018, 2020; Bendre et al. 2018; Ziegler and Domschke 2018). Notably, the direction of methylation alterations (hyper- or hypo-methylation) associated with these conditions varies and differs by proposed precipitating factors such as exposure to adversity and treatment (Bendre et al. 2018; Checknita et al. 2018, 2020; Ziegler and Domschke 2018). Thus, extant evidence suggests that methylation of the *MAOA* ROI may vary across different disorders and be highly dynamic in response to environmental factors (Ziegler and Domschke 2018).

## The present study

The study aimed to: (1) characterize methylation levels in *MAOA* ROI CpGs by age, sex, and by the interaction of age and sex, adjusting for genotype and substance dependence; and (2) determine whether *MAOA* ROI methylation levels were associated with the interaction of MAOA-uVNTR genotype, sex, and sexual abuse among males and/or females. Methylation in the *MAOA* ROI was assessed in five ways: individual CpGs; mean levels across the ROI; mean levels within intronic and exonic regions, since promotor and first exon methylation associates with suppressed gene expression, while intronic and gene body methylation associates with elevated gene expression (Brenet et al. 2011; Jones 2012; Moore et al. 2013); empirically derived components of CpG methylation levels (since we have previously found that these components vary by sex Bendre et al. 2018; Checknita et al. 2020). Participants were 252 women, mean age 33.2 (SD = 11.98) years, range 15–62 years, and 157 men, mean age 36.3 (SD = 14.48) years, range 14–73 years at the time of saliva collection for DNA extraction. Only 23 participants were less than 18 years old. Most of the sample was recruited at an outpatient clinic for adolescents misusing substances, and included former clients, their siblings, and parents. Given the nature of the sample, analyses were undertaken to determine whether substance dependence affected associations of sex and age with methylation levels of the *MAOA* ROI. Furthermore, analyses of the associations of age and sex with methylation were adjusted for several factors previously related to *MAOA*, including depression and anxiety disorders (Booij et al. 2015; Nilsson et al. 2018; Ziegler and Domschke 2018; Checknita et al. 2020), and impulsivity that characterizes individuals with antisocial disorders (Pavlov et al. 2012; Comai et al. 2012; Booij et al. 2015). Among males only, analyses were also adjusted for tobacco use that has been shown to impact methylation levels (Philibert et al. 2010).

Methylation was measured in DNA extracted from saliva. Knowledge of *MAOA* ROI methylation derives from studies sampling both blood (Philibert et al. 2008; Checknita et al. 2015; Ziegler et al. 2016) and saliva (Melas et al. 2013; Melas and Forsell 2015; Bendre et al. 2018; Checknita et al. 2018, 2020). The concordance of *MAOA* ROI methylation levels across peripheral tissues and with central tissue has not yet been exhaustively assessed. Methylation of the *MAOA* ROI in blood has also been reported to be inversely correlated with MAO enzymatic activity in the brain of adult men (Shumay et al. 2012). As such, evidence supports the use of saliva to measure methylation of the *MAOA* ROI.

We previously found that MAOA-uVNTR and sexual abuse, but not their interaction, were each associated with higher methylation of the *MAOA* ROI and current diagnosis of depression in a smaller sample of the young women included in the present study (Checknita et al. 2020). The association of sexual abuse with methylation of the *MAOA* ROI was robust to adjustment for psychoactive medication, alcohol and drug dependence, and current substance use. Given our previous findings (Bendre et al. 2018; Checknita et al. 2020) and current hypotheses that vulnerability to epigenetic changes varies by genotype (Bendre et al. 2018; Checknita et al. 2020), the associations of sex and age with methylation of the *MAOA* ROI were adjusted for genotype. Genetic polymorphisms may modify the binding affinity of methyl groups to DNA in regions up to 5000 bp downstream (Ahsan et al. 2017; Ek et al. 2018), thus contributing to the facilitation or attenuation of epigenetic modifications to genes. Furthermore, evidence from epigenome studies showed that associations of methylation with sex and age varied by region depending on genotype and environmental factors (Van Dongen et al. 2016).

## Method

### Participants

**Fig. 1:**
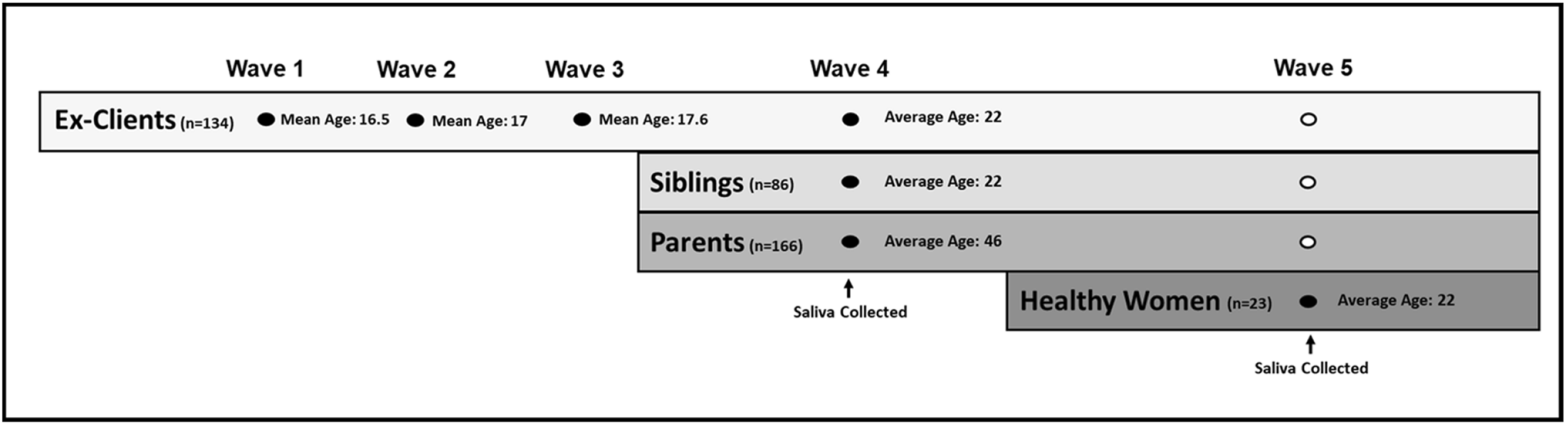
Study timeline. At waves 1 through 3, male and female ex-clients recruited in adolescence completed interviews and questionnaires. At wave 4, both ex-clients, siblings, and their parents completed interviews and questionnaires and provided saliva samples for DNA extraction. At wave 5, healthy women matched on age to the other women completed interviews and questionnaires and provided saliva samples for DNA extraction. Filled in dots represent time-points at which data were used in the current study and empty dots represent time-points at which data were not used in the current study to ensure consistency between the timing of molecular and clinical observations

The sample included 409 participants: 134 had been recruited when they were adolescents seeking treatment for substance misuse; 86 of their siblings; 103 of their mothers; 63 of their fathers; and 23 healthy women of similar age and birthplace as the ex-clients. The 134 ex-clients completed structured, validated, diagnostic interviews and questionnaires to report on substance misuse at first contact with the clinic and 6, 12, and 60 months later. At the 60 month follow-up, the siblings and parents completed similar assessments, and all 409 participants provided saliva samples for DNA extraction. At the 75 month follow-up, healthy female participants were recruited, completed assessments similar to those completed by the other participants, and provided saliva for DNA extraction. The study timeline is summarized in Fig. 1. Age was calculated as the chronological age at the time of saliva collection. Sex was coded as male or female. DNA was extracted with a standard in-silica based method from saliva samples collected with the Oragene Self-Collection Kit (DNA Genotek Inc. Ottawa, Ontario, Canada) according to the manufacturer’s guidelines. Additional details about the processing of samples are outlined in Supplementary Material.

### Measures

Genotyping and methylation procedures were performed in a blinded manner and included samples from all waves and study subgroups to mitigate potential batch effects.

#### Methylation of the *MAOA* ROI

**Fig. 2:**
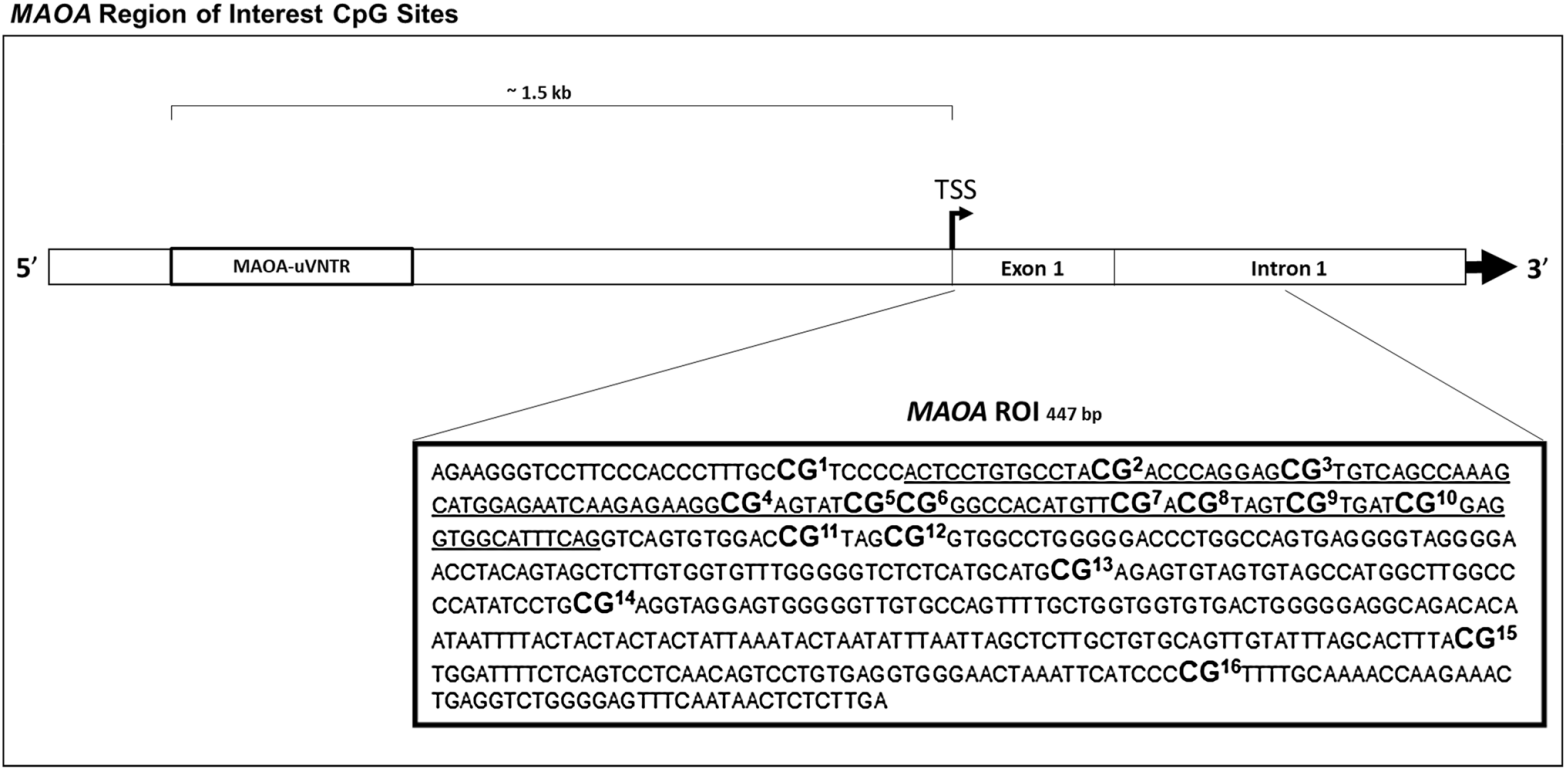
Genomic sequence of the *MAOA* ROI with the 16 CpGs included (numbered) and coding region (underlined) relative to its genomic location relative to the MAOA-uVNTR polymorphism, transcription start site (TSS), and first exonic and intronic regions of the gene. Genomic locations illustrated are scaled for interpretive ease

Methylation analysis targeted a previously characterized 448 bp ROI (hg19 chrX: 43,515,544—43,515,991) comprised of 16 CpGs spanning the first exon and part of the first intronic region of *MAOA* (Shumay et al. 2012; Checknita et al. 2015). Genomic DNA extracted from saliva was first bisulfite-treated using EZ DNA Methylation™ Kit (Zymo Research Corporation, Irvine, California) and then assayed using Sequenom’s EpiTYPER at Karolinska Institutet’s Mutation Analysis Core Facility (MAF). Resulting data represented the percentage of methylation at each CpG to the nearest 0.5%. The 16 CpGs were denoted numerically based on their 5´–3´ position within the ROI based on the forward strand genomic sequence. To optimize technical outcomes, an amplicon designed on the reverse strand covered CpGs 1–13 and another amplicon covering CpGs 13–16 was designed on the forward strand. In addition to the 16 individual CpGs, variables for mean levels of methylation for genomic features (exonic CpGs 2–10, and intronic CpGs 11–16), and overall ROI (all CpGs) were calculated. The *MAOA* ROI genomic sequence is provided in Fig. 2.

#### Genotyping of the MAOA-uVNTR

Genotyping was performed using a standard PCR technique, followed by gel electrophoresis. The target 30-bp repeat target region of *MAOA* (MAOA-uVNTR) was amplified using forward primer 5' ACA GCC TGA CCG TGG AGA AG 3' and reverse primer 5' GAA CGG ACG CTC CAT TCG GA 3' (Sabol et al. 1998). In accordance with prior in-vitro functional analyses of the MAOA-uVNTR (Beach et al. 2010), the three repeat variants were denoted as the short (MAOA-S) allele, and 3.5, 4, or 5 repeat variants as the long (MAOA-L) allele in men. In women, *MAOA* genotype was denoted as MAOA-SS or MAOA-LL in homozygous women and MAOA-SL in heterozygous women. Hardy–Weinberg Equilibrium for MAOA-uVNTR genotype was confirmed using an *X*^2^ test among women (*p* = 0.29) but not men since distribution of genotype is the same as the allelic distribution. Furthermore, the allelic frequencies were similar to those reported in other studies including samples of Swedish men and women (Nilsson et al. 2006; Sjoberg et al. 2007; Åslund et al. 2011).

#### Lifetime history of mental disorders

Clinicians administered the Structured Clinical Interview for DSM-IV (SCID I) (First et al. 2002) to all participants at the time of saliva collection and, to most, also at baseline. Lifetime diagnoses of alcohol and/or drug dependence were acquired by 24.3% (*n* = 61) women and 34.2% (*n* = 53) men (*X*^2^(2,*N* = 406) = 4.64 *p* = 0.040). Lifetime diagnoses of depression disorders (major depression disorder, dysthymia, depression disorder not-otherwise-specified, or substance-induced mood disorder) were observed in 58.2% (*n* = 146) women and 42.6% (*n* = 66) men (*X*^2^(2,*N* = 406) = 9.33 *p* = 0.040). Lifetime diagnoses of anxiety disorders (agoraphobia, generalized anxiety disorder, anxiety disorder not-otherwise-specified, obsessive compulsive disorder, panic disorder, post-traumatic stress disorder, social phobia, specific phobia, or substance-induced anxiety disorder) were observed in 48.2% (*n* = 121) women and 31.0% (*n* = 48) men (*X*^2^(2,*N* = 406) = 11.72 *p* = 0.001). The impulsivity symptom of antisocial personality disorder characterized 2.5% (*n* = 6) women and 9.9% (*n* = 15) men (*X*^2^(2,*N* = 396) = 10.24 *p* = 0.002).

#### Tobacco use

At the time of DNA collection, male ex-clients and siblings reported on use of cigarettes and snus (a small bag of dried snuff tobacco placed under the lip). Responses were coded as: (0) “No”, and (1) “Yes” if any smoking and/or use of snus were reported. Among these males, 42.0% (*n* = 66) reported tobacco use.

#### Sexual abuse

Information on sexual abuse was only available for the ex-clients, siblings, and healthy women (*n* = 243). These participants reported on sexual abuse at all waves of data collection using items from the Sexual Experience Survey (Koss and Oros 1982), Sexual and Physical Abuse Questionnaire (Kooiman et al. 2002), and McArthur Community Violence Instrument (Steadman et al. 1998). Sexual abuse was coded as absent (0) or present (1) if any of the following were reported as occurring at any time: forced to engage in sexual activity against his or her will by a person in authority, by offering alcohol or drugs, or by physical violence. Among the women, 43.8% (*n* = 64) reported having experienced sexual abuse as did 14.4% (*n* = 13) of the men (*X*^2^(2,*N* = 236) = 21.88 *p* < 0.001).

### Statistical analyses

#### Characterization of *MAOA* ROI methylation by sex and age

The first set of analyses aimed to provide a cursory descriptive analysis of methylation levels in our study sample. The number of participants included in these models was varied slightly due to missing data as not all participants had useable methylation data for all CpGs, *MAOA*-uVNTR genotypes were missing for five participants, substance dependence diagnoses were missing for three participants, and reports of sexual abuse were missing for seven participants. Associations of sex and age with methylation of each CpG within the *MAOA* ROI, across the ROI, of intronic and exonic regions, and of the empirically derived components of CpG methylation were estimated. To identify homogenous groups (components) of methylation within the *MAOA* ROI, Principal Component Analyses (PCA) of methylation of the 16 *MAOA* ROI CpGs with Varimax rotation and Kaiser normalization were computed, first among all 409 participants and then for women and men. Pearson correlations were used to verify associations between component methylation and the mean methylation levels of the CpGs included in the component.

Participants’ ages were bimodally distributed. An age-group variable was constructed: young with a mean of 22.7 (SD = 3.61) years; and old with a mean of 46.1 (SD = 7.02) years. Young and old participants were compared on mean methylation levels at each CpG within the *MAOA* ROI and across the ROI using a two-way mixed-model ANOVA with post hoc Bonferroni corrections for multiple comparisons, on methylation of the intronic and exonic regions, and the homogeneous components of methylation using independent sample *t* tests.

Women and men were compared on mean methylation levels at each CpG within the *MAOA* ROI and across the ROI using a two-way mixed-model ANOVA with post hoc Bonferroni corrections for multiple comparisons, and on methylation of the intronic and exonic regions and components of methylation using independent sample *t* tests.

To determine if age moderated associations of sex with methylation levels of individual CpGs, across the ROI, of intronic and exonic regions, and of homogeneous components, PROCESS 2.16 for SPSS was used with the simple moderation modeling procedure outlined by Hayes (2013). Separate models were computed for levels of individual CpGs, across the ROI, of intronic and exonic regions, and of homogeneous components of methylation as dependent variables (Y). Sex was entered as the independent variable (X), and age (a continuous variable) was entered as the moderating variable (M). Conditional effects (“simple slopes”) of the moderator, age, on associations between sex and methylation levels were used to interpret significant interaction effects. The conditional effects procedure subdivided age using Mean ± 1SD criteria; low (Mean − 1SD, age = 19), moderate (Mean, age = 32), and high (Mean + 1SD, age 45). Significant models were re-run adjusting for *MAOA* genotype.

Next, we examined associations between age as a continuous variable and methylation separately by sex among the 252 women and 157 men. The sex-specific PCA analyses were performed to identify methylation components. Linear regression models were computed to examine associations of age in years with methylation of individual CpGs, across the ROI, of intronic and exonic regions, and of sex-specific components of methylation. Significant regression models were subsequently adjusted for MAOA-uVNTR genotype.

Because a large proportion of participants had a history of substance misuse, and some substance misuse at the time of DNA extraction, all linear regression analyses that were computed separately by sex to examine the association of age with methylation of individual CpGs, across the ROI, of exonic and intronic regions, and of sex-specific components were re-run adjusting for lifetime diagnoses of substance dependence. Similar adjustments for depression disorders, anxiety disorders, impulsivity, and tobacco use were computed for methylation of the ROI, exonic and intronic regions, and sex-specific components.

#### Examining the interaction of sex, MAOA-uVNTR genotype, and sexual abuse on methylation

To determine whether sex would continue to be associated with methylation of the intronic and exonic regions and the sex-specific components of methylation within the *MAOA* ROI when taking account of genotype (females SL and LL versus SS), sexual abuse, and their interactions, four General Linear Models were computed. These analyses were again done separately by sex. Parents were not included in these analyses as they had not reported on their own experiences of sexual abuse. Furthermore, the analyses were also run including ex-clients and healthy participants and excluding siblings.

## Results

### Characterization of *MAOA* ROI methylation by sex and age

#### Analysis in the full sample

##### Empirically derived components of methylation

As presented in Table 1, among the 409 participants, principal component analysis of methylation across the 16 CpGs in the *MAOA* ROI identified two components based on Eigen values over Kaiser’s criterion of 1; (1) CpGs 2–14, and (2) CpGs 15 and 16, accounting for 69.96% and 17.22% of the variation in methylation levels, respectively. Pearson’s correlations revealed that component methylation was highly correlated with the mean value of its constituent CpGs (component 1, *r* = 0.992, p < 0.001; component 2, *r* = 0.961, *p* < 0.001).

**Table 1:** Correlation coefficients for rotated component matrix from principal component analysis of *MAOA* ROI CpG methylation in all participants, women only, and men only

Component		CpG Site											
		CpG 2/3		CpG 4	CpG 5/6	CpG 7/8	CpG 10	CpG 11	CpG 12	CpG 13	CpG 14	CpG 15	CpG 16
All participants													
1	0.959		0.956		0.964	0.959	0.966	0.965	0.874	0.735	0.767	–	–
2	–		–		–	–	–	–	–	–	–	0.908	0.841
Women													
1	0.895		0.831		0.904	0.863	0.933	0.895	0.590	–	–	–	–
2	–		–		–	–	–	–	–	0.641	0.711	0.845	0.670
Men													
1	0.872		0.795		0.882	0.861	0.848	0.788	0.482	–	–	–	–
2	–		–		–	–	–	–	–	0.847	0.607	0.895	0.927

##### Sex

**Fig. 3:**
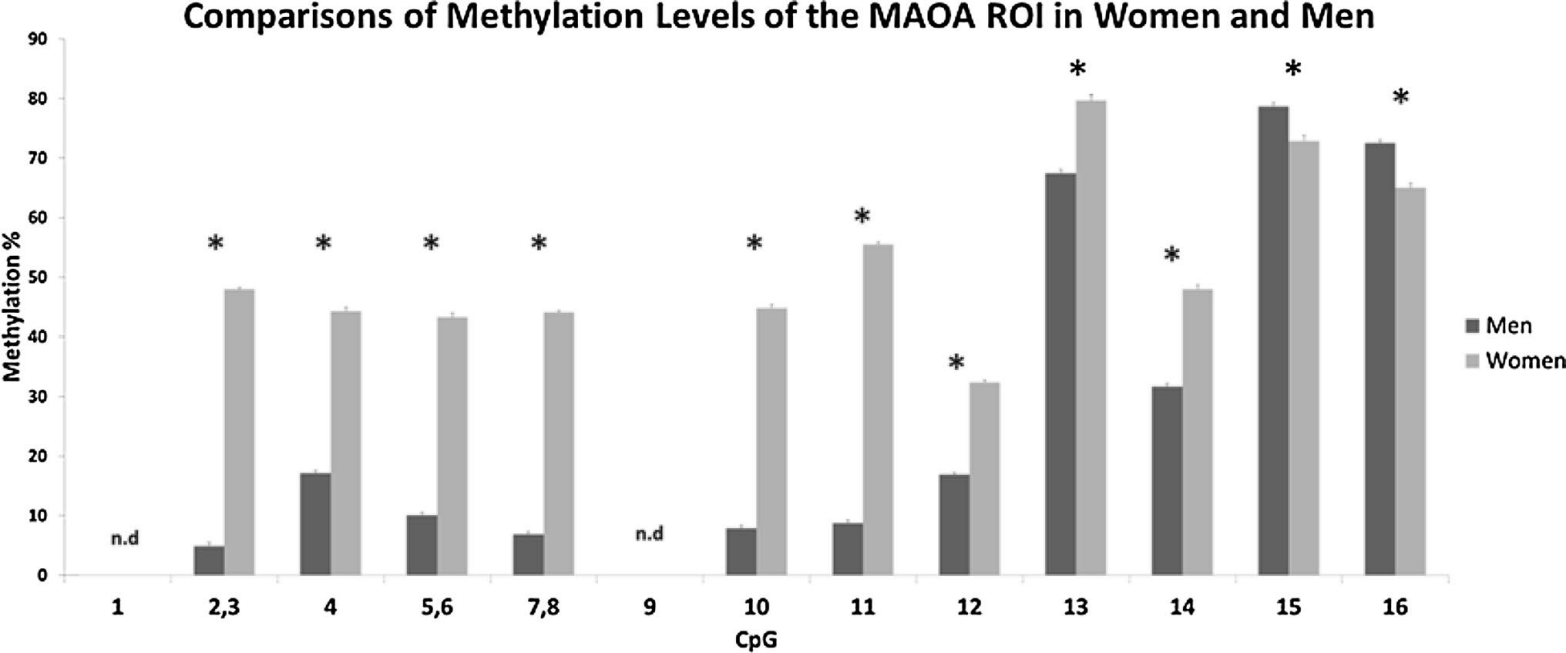
Results of comparisons of men and women on *MAOA* ROI CpG methylation. (*p < 0.05) (n.d: No data)

A two-way mixed-model ANOVA examining associations of sex and CpG methylation and overall methylation indicated that women displayed higher levels of ROI methylation than men, with post hoc Bonferroni analyses showing that women had higher methylation levels of CpGs 2–14, while men displayed higher methylation levels of CpGs 15 and 16. Results are illustrated in Fig. 3. Independent samples *t* tests indicated that women displayed higher exonic (*t*(409) = 53.21, *p* < 0.001), and higher intronic (*t*(409) = 19.95, *p* < 0.001) methylation levels than men. Independent samples *t* tests also indicated that women showed higher component 1 methylation levels than men (*t*(409) = 47.91, *p* < 0.001), and that men showed higher component 2 methylation levels than women (*t*(409) = 4.21, *p* < 0.001). Thus, methylation levels of individual CpGs within the *MAOA* ROI, in intronic and exonic regions, in components of methylation differed by sex. Mean methylation levels in men and women for all CpGs, the exonic and intronic regions, overall *MAOA* ROI, and components 1 and 2 are provided in Table S1. Sex-wise comparisons of all CpG, exonic and intronic, overall *MAOA* ROI, and component 1 and 2 methylation levels by *MAOA* genotypes are provided in Table S2.

##### Age-group

Results of a two-way mixed-model ANOVA detected no differences by age-group of methylation levels of individual CpGs or methylation across the ROI. Independent sample *t* tests revealed no differences by age-group in exonic and intronic regions or with the empirically derived components. Thus, age-group, older or younger, was not associated with various measures of methylation of the *MAOA* ROI.

#### Are the associations of sex with methylation levels modified by age?

Age moderated the association of sex with methylation levels of CpG15 (*B* = − 0.001, *p* = 0.024). Follow-up analyses revealed significant conditional effects, such that women at mean (age = 32) and mean + 1SD (age = 45) years exhibited lower methylation levels of CpG15 than men. Only sex was associated with methylation levels of all other CpGs (results are summarized in Table S3). Significant conditional effects are summarized in Table 2. Only sex was significantly associated with methylation levels overall in the ROI methylation and within intronic and exonic regions, such that women showed higher levels than men.

**Table 2:** Conditional effects (“Simple Slopes”) of sex and age on *MAOA* ROI methylation among women compared to men

Age	Unstandardized simple slope (beta coefficient)	95% confidence interval	*p*
Component 2 Methylation (CpGs 15 and 16)			
1 SD below mean^a^	− 0.2269	-0.51 to 0.06	0.120
Mean^b^	− 0.4276	− 0.63 to − 0.22	<0 .001
1 SD above mean^c^	0.5997	− 0.91 to − 0.35	< 0.001
CpG15 methylation			
1 SD below mean^a^	− 0.0311	− 0.06 to 0	0.051
Mean^b^	− 0.0556	− 0.08 to − 0.03	< 0.001
1 SD above mean^c^	− 0.0801	− 0.11 to − 0.05	< 0.001

^a^1 SD below mean (age = 19)

^b^Mean (age = 32)

^c^ SD above mean (age = 45)

The interaction of age (a continuous variable) and sex with component 1 methylation levels was not significant (*B* = − 0.003, *p* = 0.370). Only sex was significantly associated with component 1 methylation levels, with women displaying higher levels than men (*B* = 1.98, *p* < 0.001). The interaction of age and sex with component 2 methylation levels was significant (*B* = − 0.015, *p* = 0.04). Follow-up analyses showed a significant conditional effect such that women at mean (age = 32) and mean + 1SD (age = 45) ages exhibited lower methylation levels of component 2 than men. Results are presented in Table 2.

Significant results were adjusted for *MAOA* genotype. Among women, the interaction of sex and age with CpG15 methylation was robust to adjustment for *MAOA* genotype (*B* = − 0.182, *p* = 0.031). By contrast, among women, the interaction of age and sex with component 2 was not robust to adjustment for genotype (*B* = − 0.014, *p* = 0.060). Furthermore, among women, the association of age and component 2 methylation was no longer significant once *MAOA* genotype was included in the model (*B* = − 0.009, *p* = 0.057).

#### Analyses in women only

##### Empirically derived components of methylation

Among the 252 women, principal component analysis of methylation across the 16 CpGs in the *MAOA* ROI identified two components based on Eigen values over Kaiser’s criterion of 1; (1) CpGs 2–12, and (2) CpGs 13–16, accounting for 49.41% and 19.14% of the variation in methylation levels, respectively. Results are presented in Table 1. Pearson correlations revealed that component methylation was highly correlated with the mean values of its constituent CpGs (component 1, *r* = 0.996, *p* < 0.001), (component 2, *r* = 0.991, *p* < 0.001).

##### Associations of methylation levels with age

Linear regression models revealed a negative association of age with methylation levels of CpG 13 (*F*(1,239) = 14.94, *p* < 0.001, *B* = − 0.21, *r*^2^ = 0.055), no association of age with overall methylation nor with methylation of the exonic and intronic regions, and a negative association of age with component 2 methylation (*F*(1,239) = 5.13, *p* = 0.024, *B* = − 0.01, *r*^2^ = 0.017). The association of age with CpG 13 methylation was robust to adjustment for *MAOA* genotype (*B* = − 0.208, *p* < 0.001), while the association of age and component 2 was not (*B* = − 0.009, *p* = 0.057).

#### Analyses in men only

##### Empirically derived components of methylation

Among the 157 men, principal component analysis of methylation across the 16 CpGs in the *MAOA* ROI identified two components based on Eigen values over Kaiser’s criterion of 1; (1) CpGs 2–12, and (2) CpGs 13–16, accounting for 44.63% and 23.63% of the variation in methylation levels, respectively (Table 1). Pearson’s correlations revealed that component methylation was highly correlated with the mean value of its constituent CpGs (component 1, *r* = 0.985, *p* < 0.001), (component 2, *r* = 0.994, *p* < 0.001).

##### Associations of methylation levels with age

Among the men, linear regressions revealed a positive association of age with CpG 15 methylation (*F*(1,150) = 4.58, *p* = 0.034, *B* = 4.15), *r*^2^ = 0.023, and no association with the methylation of the other CpGs or across the ROI. Age was not significantly associated with methylation of intronic and exonic regions, nor with components of methylation. The association of age with CpG 15 methylation was robust to adjustment for *MAOA* genotype (*B* = − 0.182, *p* = 0.031).

A summary of full sample and sex-specific results are presented in Table 3.

**Table 3:** Associations of age and sex with methylation levels of the MAOA-ROI: a summary of results

	CpGs	Across ROI	Intronic	Exonic	Component 1	Component 2
All participants						
Age-group	x	x	x	x	x	x
Sex	↑women CpG 2–14 ↓women CpGs 15 and 16	↑women	↑women	↑women	↑women	↓women
Sex modified by age	In women, at CpG 15^B^					In women^A^
Only sex		↑women	↑women	↑women		
Women only						
Age	Negative association at CpG 13^B^	x	x	x	x	Negative association^A^
Men only						
Age	Positive association at CpG 15^B^	x	x	x	x	x

A. Not robust to covariation with MAOA-uVNTR genotype

B. Robust to adjustment for MAOA-uVNTR genotype

x. No association

### Are associations of sex and age with methylation levels modified by substance dependence, depression disorders, anxiety disorders, impulsivity, or tobacco use?

Given that most of the sample included adolescents presenting with substance misuse, their siblings and parents, analyses were re-run with lifetime substance dependence as a covariate. Results of all linear regression models among women were robust to adjustments for lifetime substance dependence, while, among men, the positive association of age with CpG15 methylation was no longer significant (*B* = 0.120, *p* = 0.068). Additionally, among men, the association of age with mean exonic methylation remained non-significant in the adjusted model, though lifetime substance dependence was positively associated with mean exonic methylation (*B* = 2.876, *p* = 0.011).

The linear regression models on genomic feature and component methylation were also re-run to determine whether lifetime depression and anxiety disorders, impulsivity, and tobacco use (in men only) modified results. Among women, depression diagnoses were associated with higher levels of exonic (*B* = 0.136, *p* = 0.034) and component 1 (*B* = 0.136, *p* = 0.035) methylation, though age remained non-significant in these models. The negative association of age with component 2 methylation levels in women was robust to adjustment for depression and anxiety disorders, and impulsivity.

Among the men, most models were robust to adjustments, although a positive association of age with component 2 methylation was observed after adjusting for tobacco use (*B* = 0.236, *p* = 0.030). While the association of age with first exon methylation levels remained non-significant in the model, a positive association with anxiety diagnoses (*B* = 0.189, *p* = 0.022) was observed.

### Is higher methylation among women associated with genotype, sexual abuse, and their interactions?

**Fig. 4:**
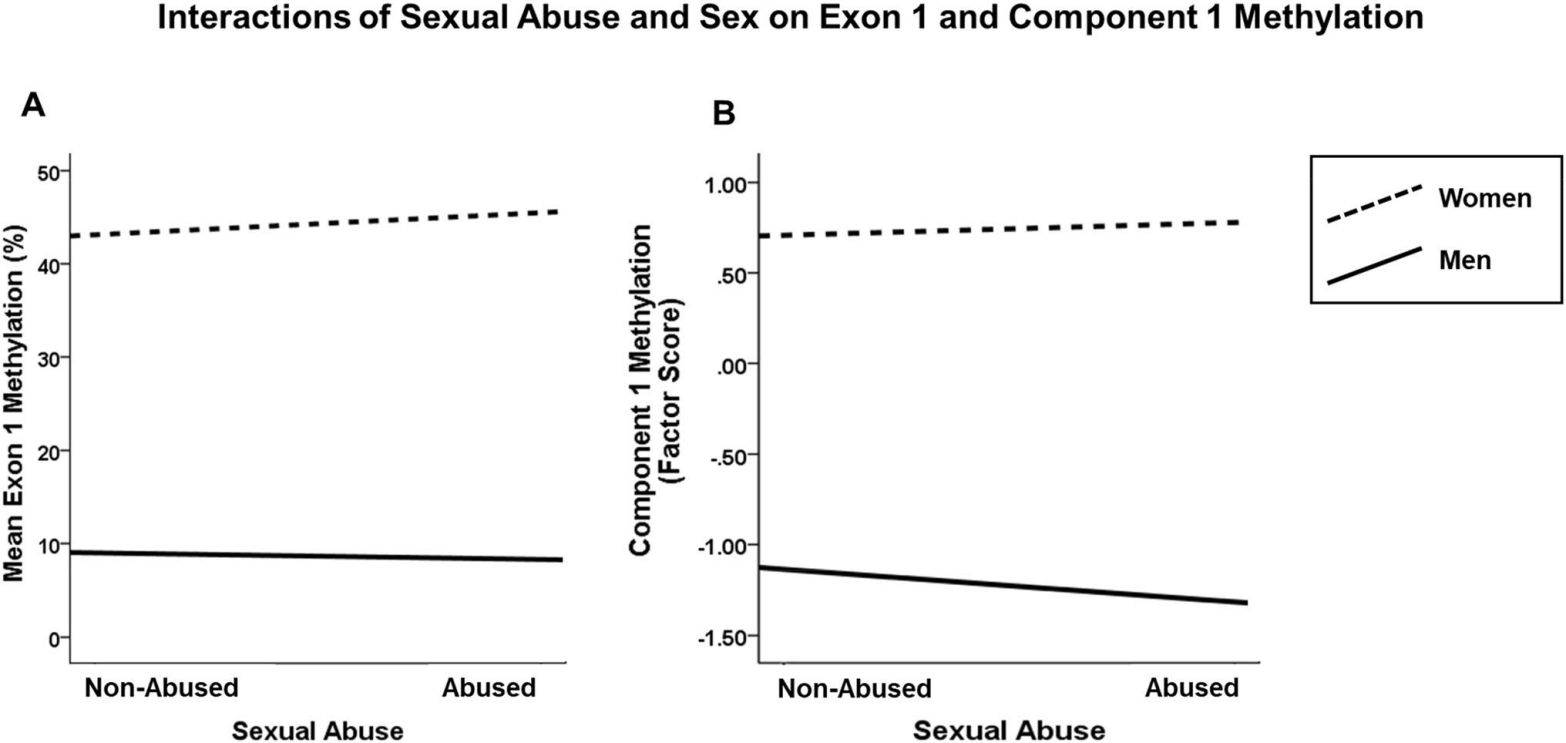
Interactions of sexual abuse status (no sexual abuse vs. sexual abuse) and sex on exon 1 methylation level (**A**) and component 1 factor score (**B**) in the young women and men (ex-clients, siblings, and healthy women)

As presented in Table 4, among the younger participants, neither genotype nor sexual abuse was directly associated with methylation in intronic and exonic regions and components. The only significant direct effect was sex on intronic, exonic, and component 1 methylation. Exonic methylation and component 1 methylation were additionally associated with the interaction of sex and sexual abuse. Intronic methylation was only associated with sex. Figure 4 illustrates the two significant interaction terms showing higher methylation levels in women than men regardless of sexual abuse. However, as recommended, we did not interpret direct effects when effects of interaction terms were statistically significant (Nelder 1998). Thus, the results indicate that sexual abuse was associated with higher methylation levels in the *MAOA* ROI among women. To further explore the association of sexual abuse with methylation levels among women and men, we compared intronic, exonic, and component methylation among abused and non-abused participants within each sex. These exploratory, descriptive analyses (Tables S4 and S5) indicate that among men, methylation levels of those who did and who did not experience sexual abuse did not differ, while among women, those who were sexually abused exhibited higher methylation in the exonic region and in component 1 than the non-abused, and lower methylation in component 2.

**Table 4:** General linear models of factors associated with *MAOA* ROI methylation in exonic and intronic regions and components 1 and 2

					Methylation							
		Exonic			Intronic			Component 1			Component 2	
	*df*	*F*	*p*	*df*	*F*	*p*	*df*	*F*	*p*	*df*	*F*	*p*
MAOA-uVNTR	1	0.302	0.583	1	0.143	0.705	1	1.501	0.221	1	0.678	0.410
Sexual abuse	1	1.510	0.219	1	1.050	0.305	1	3.688	0.055	1	1.363	0.243
Sex	1	1747.654	< 0.001	1	215.215	< 0.001	1	1294.844	<0 .001	1	2.414	0.120
Sexual abuse × Sex		4.161	0.041		1.088	0.297		4.973	.026		0.931	0.335
MAOA-uVNTR × Sexual Abuse		1.045	0.307		2.390	0.122		3.092	.079		1.523	0.217
MAOA-uVNTR × Sex		0.559	0.455		0.036	0.850		1.281	.258		0.359	0.549
MAOA-uVNTR × Sexual Abuse x Sex		1.223	0.269		3.152	0.076		3.590	.058		2.269	0.132

To mitigate the impact of including related siblings in these analyses, the models were re-run among only the ex-clients and healthy participants (no siblings). Results of the models are summarized in Table S6. Notably, results are very similar to those obtained with the larger sample. Again, the only significant direct effects were the association of sex with intronic, exonic, and component 1 methylation. Only exonic methylation was associated with the interaction of sex and sexual abuse. Unlike results from the larger sample, component 1 methylation was associated with the interaction of MAOA-uVNTR genotype and sex. Further exploration showed that women who had experienced sexual abuse displayed higher exonic methylation than those who did not (*p* = 0.017). Among men, no difference in exonic methylation was observed between men who did and did not experience sexual abuse (*p* = 0.978).

## Discussion

The first aim of the current study was to characterize associations of sex and age with methylation in a region-of-interest (ROI), spanning the first exonic region and partial first intronic region of *MAOA*. Methylation levels of individual CpGs, across the entire ROI, in exonic and intronic regions, and in homogeneous empirically derived components of CpG methylation were measured. Women were characterized by higher methylation levels than men and varied little by age. The only previous study to examine sex differences in methylation of *MAOA* included a small sample and observed higher levels among women than men in the exonic region (Melas and Forsell 2015). The present study confirmed this finding in a larger sample and showed higher levels of methylation within the *MAOA* ROI among women than men and in the intronic region. These findings are consistent with the results of epigenome-wide methylation studies (Nugent and McCarthy 2011). Additionally, the present study extends knowledge by showing that within the *MAOA* ROI, methylation levels among women and men generally did not decrease with age. This finding is consistent with epigenome-wide studies, indicating that methylation levels in some regions (Ciccarone et al. 2018), particularly those in first exon/intron junctions (Kochmanski and Bernstein 2020), are more likely than other regions to remain open to fluctuation in spite of global reductions in methylation.

Among women, results of the moderation analyses indicated that the interaction of sex and age with methylation at CpG 15 was robust to adjustment for MAOA-uVNTR genotype, and genotype dependent at component 2. Similarly, among women, the association of age with methylation at CpG 13 was robust to adjustment by genotype, whereas the association of age with component 2 was no longer significant after adjustment for genotype. Among men, the one direct association of age with methylation of CpG 15 was robust to adjustment for genotype. As such, the few associations of age with methylation identified in the women and men were largely independent of MAOA-uVNTR genotype. Following adjustments for lifetime history of substance dependence, depression disorders, anxiety disorders, impulsivity, and tobacco use (only in men), findings were largely unchanged with age showing few associations with methylation levels.

The second aim of the study was to determine whether *MAOA* ROI methylation levels were associated with the interaction of MAOA-uVNTR genotype, sex, and sexual abuse among males and/or females. Analyses that included only the younger participants revealed that methylation levels were not associated with genotype, nor with the interaction of genotype and sexual abuse, and were slightly increased among survivors of sexual abuse. In fact, the association of methylation levels within the *MAOA* ROI with a negative environmental factor—sexual abuse—was much weaker than the association with sex. Furthermore, in these models, sex remained the factor most strongly associated with intronic, exonic, and component 1 methylation levels even after taking account of MAOA-uVNTR genotype, sex, and sexual abuse. Exonic and component 1 methylation were associated with the interaction of sex and sexual abuse. To ensure that the inclusion of siblings had not confounded these results, models were re-run with only unrelated ex-clients and healthy participants. Results were similar with those in the larger sample, showing that sex was the factor most strongly associated with exonic, intronic, and component 1 methylation levels, while exonic levels were also associated with the interaction of sex and sexual abuse. Notably, in these models, there were no direct associations of MAOA-uVNTR genotypes with methylation levels. Thus, even when taking account of MAOA-uVNTR genotypes and sexual abuse, and the interactions of these factors, sex remained the principal factor associated with methylation levels observed within the *MAOA* ROI. Despite the small sample size, and the low number of sexually abused males, results suggested that sex was associated with the magnitude of the difference in methylation levels among participants who had and had not experienced sexual abuse. The finding that these associations were not dependent on genotype is consistent with results from a recent study, showing that genetic factors associate with stability of methylation levels, while environmental factors associate more strongly with alterations to methylation (Reynolds et al. 2020).

Considered together, our findings within the *MAOA* ROI are consistent with prior evidence of sex differences in methylation across the genome (Yousefi et al. 2015; Ratnu et al. 2017; Suderman et al. 2017), and extend knowledge by showing that within the *MAOA* ROI women present higher methylation levels than men for approximately 3 decades following puberty. Previous evidence of a decrease in methylation levels across the genome as individuals age (Ciccarone et al. 2018) was not observed in the *MAOA* ROI, except in intronic CpG13 and component 2 among women. Perhaps, the age range of the participants—approximately 3 decades following puberty—was insufficient to identify age-related changes or did not cover critical periods of change. Alternatively, studies have reported that methylation in first exonic–intronic junctions such as the *MAOA* ROI, relative to other regions, is more likely to be spared from the global pattern of demethylation as individuals grow older (Ashapkin et al. 2017; Ciccarone et al. 2018; Kochmanski and Bernstein 2020). Perhaps, through adulthood, these regions remain open to fluctuation in response to environmental and biological factors which contribute to health-related outcomes (Ashapkin et al. 2017; Ciccarone et al. 2018). However, as the results examining sexual abuse showed, this may be the case more often among women than men. This interpretation warrants further investigation as the present sample included few males who had experienced sexual abuse.

As EpiTYPER samples methylation randomly from both X-chromosomes in females, the resulting levels represent the averaged level of methylation from both chromosomes (Cotton et al. 2011). As such, the large sex difference, particularly in the exonic region of the *MAOA* ROI, likely indicates near total methylation from the inactivated chromosome and low methylation from the active chromosome, thus accounting for exonic methylation levels in in the ~ 40–50% range among women (Table S1). Similar levels of *MAOA* first exon methylation in women have been reported and interpreted similarly by others examining methylation in this region (Melas et al. 2013; Melas and Forsell 2015; Ziegler et al. 2016). As the sex difference in methylation levels in the *MAOA* first exon remained after taking other factors into account, our findings offer further evidence that the *MAOA* first exon may be an X-inactivation site in women. The finding that methylation of first exonic regions of genes is associated with total transcriptional silencing of a gene rather than downregulated transcription (Brenet et al. 2011) may indicate that *MAOA* is not among the ~ 15% of X-linked genes that escape inactivation (Carrel and Willard 2005). *MAOA* expression may thus be monoallelic rather than biallelic in women. Given the consistency of the sex difference in methylation levels reported in the current and previous studies (Melas et al. 2013; Melas and Forsell 2015), it is highly likely that it is due to X-inactivation.

Other factors could influence sex differences in *MAOA* methylation on the active X-chromosome in women. Some evidence suggests that *MAOA* may be a genomic locus where sex hormones influence serotonergic activity and phenotypes that feature prominently in our study sample, such as antisocial behaviour and substance misuse (Nilsson et al. 2018). For instance, one study found that the interaction of *MAOA* genotype and testosterone concentration in cerebrospinal fluid predicted antisocial behaviour and suggested that this interaction may have been mediated by direct effects on *MAOA* transcription (Sjöberg et al. 2009). Another study of healthy males showed that those carrying MAOA-S, as compared to those carrying MAOA-L, displayed increased risk-taking behaviours following administration of a topical gel containing testosterone (Wagels et al. 2017). Furthermore, *MAOA* expression may be modified by interactions between testosterone levels and transcription factor Sp1 at its binding sites throughout the *MAOA* promoter region (Ou et al. 2006). Given that DNA methylation can modify gene expression by disrupting transcription factor binding (Bird 2011), the impact of sex hormones on *MAOA* expression may be influenced by epigenetic processes. Although potential influences of female sex hormones on *MAOA* expression and serotonergic activity in such phenotypes remain unclear (Booij et al. 2015; de Almeida et al. 2015; Raine 2019), investigations of such associations will be important to further elucidate sex differences in *MAOA* regulation.

### Limitations and strengths of the present study

Limitations of the study included the EpiTYPER method that is unable to distinguish between DNA methylation and 5-hydroxymethylation, which is a further modification of a methylated cytosine through enzymatic oxidation and a proposed marker of DNA demethylation processes (Kumar et al. 2018). DNA 5-hydroxymethylation may also have unique functional impacts on gene transcription that are distinct from those conferred by methylation, though these functional impacts are not yet fully understood (Zheleznyakova et al. 2016). In addition, genomic regions spanning exon–intron boundaries (Ciccarone et al. 2018), such as the *MAOA* ROI, show enrichments of hydroxymethylation (Kochmanski and Bernstein 2020). This could be one reason why no reduction in methylation levels with increasing age was detected. As such, it is vital for future studies of the *MAOA* ROI to examine the ratio of DNA methylation to 5-hydroxymethylation, their association with age, and their respective contributions to *MAOA* transcriptional activity.

Our study sample was relatively small, thus providing insufficient power to perform multiple corrections for all statistical models, particularly in the models that included direct effects, and two- and three-way interaction terms to determine associations with exonic, intronic, and component methylation levels. However, based on previous methylation studies (Jones 2012; Moore et al. 2013), these regions were regarded as distinct functional units, greater than their constituent CpG sites, in terms of impact on gene expression, thus making corrections for multiple testing in these models unnecessary. The sample sizes often recommended to examine gene-by-environment interactions stem from earlier critiques, suggesting that the effect sizes of interactions should exceed those for a candidate gene as detected by genome-wide association studies including large sample sizes (Duncan and Keller 2011). However, it has been more recently proposed that there is likely no main effect of *MAOA* genotypes independent from the influence from environmental factors (Booij et al. 2015; Åslund and Nilsson 2018; Nilsson et al. 2018). If an interaction term is associated with a dependent variable, the main effect becomes difficult to interpret (Nelder 1998; Nilsson et al. 2014), particularly since the main effect will change with any additional variables entered into the interaction (Nilsson et al. 2018). The initial comparisons of methylation levels by age and by sex were made using two-way mixed-model ANOVA which included CpG as a repeated measure. These analyses estimated group differences in overall methylation and differences at each CpG using stringent Bonferroni corrections for multiple comparisons. As such, this approach offered a means to mitigate issues with multiple testing, as has been used in prior epigenetic studies (Labonte et al. 2012; Gross et al. 2013; Cruceanu et al. 2016).

Statistical analyses of X-linked genes in GWAS are typically conducted within sex (Weir et al. 2006; Sillanpää 2011). GWAS typically adjust for relatedness of participants. To address these considerations, we assessed methylation levels both in the full sample and separately by sex and re-ran models that examined interactions of sex, sexual abuse, and genotype including only unrelated participants. Similar exclusions were not used in the first set of analyses aimed at providing a cursory characterization of methylation levels in our study sample.

However, while the sample was small and had limited statistical power, it was well characterized by multiple face-to-face interviews and questionnaires. Some authors have suggested that studies of gene-by-environment interactions in smaller samples may be preferable as dependent variables are more likely to be assessed using in-person interviews with better reliability and validity than the telephone, post, or Internet-based assessments used in studies with large samples (Uher and McGuffin 2008; Caspi et al. 2010; Karg et al. 2011; Moffitt and Caspi 2014; Nilsson et al. 2018). The use of such in-person measures is a key strength of the current study.

Another strength of the study was the use of five different measures of methylation levels, including a novel approach, principal component analyses, to identify components of CpG with similar and distinct methylation levels. The use of component analyses also helped mitigate, to some extent, concerns regarding the low statistical power by reducing the number of variables included in our interaction analyses. Another strength of the study were the analyses conducted to confirm differences in levels of methylation in exonic regions and component 1 between women who had, and who had not, experienced sexual abuse. Our study also benefited from the use of gold-standard genotyping.

As few males in our sample had experienced sexual abuse (*n* = 13), the lack of association between sexual abuse and methylation of the *MAOA* ROI observed should be interpreted cautiously, particularly since evidence indicates that males are particularly susceptible to the impacts of adversity in both biological and behavioural consequences (Raine 2019). One previous study by our group in an overlapping sample of these men showed that, while maltreatment defined as having experienced physical and/or sexual abuse was not directly associated with *MAOA* ROI methylation levels, the interaction of maltreatment, the MAOA-S genotype, and *MAOA* ROI methylation was associated with increased aggressive behavior (Checknita et al. 2020) and alcohol consumption (Bendre et al. 2018). As such, conclusions regarding associations of maltreatment and *MAOA* ROI methylation in men may require more nuanced interpretations including other genetic and environmental factors and framed in the context of different outcomes.

We examined methylation of DNA extracted from peripheral cells in saliva, thus limiting the interpretation of our results in relation to brain processes as methylation patterns are often tissue-specific (Deaton and Bird 2011). However, the current evidence shows associations of *MAOA* ROI methylation in whole blood DNA with MAO-A enzymatic activity in the brain. Furthermore, in-silico cross-tissue examination of two available CpGs within the *MAOA* ROI using IMAGE-CpG (http://han-lab.org/methylation/ default/imageCpG#) (Braun et al. 2019) showed a fairly strong association between methylation levels from blood and saliva samples at these two CpGs with rho values of 0.64 and 0.79, respectively, and between brain and saliva methylation with rho values of 0.76 and 0.69, respectively. The methylation levels reported for the male and female participants in our study are similar to those reported elsewhere in whole blood samples from men (Checknita et al. 2015; Ziegler et al. 2018) and women (Ziegler et al. 2016; Schiele et al. 2018) and in studies examining salivary methylation of this region in men and women (Melas et al. 2013; Melas and Forsell 2015). Recent reviews in behavioural epigenetics emphasize that the value of investigations of methylation in peripheral tissues is critical for the study of mental and behavioural disorders and responses to trauma (Labonte and Turecki 2012; Lutz and Turecki 2014; Szyf 2015). As such, the extensive characterization of methylation of the *MAOA* ROI provided in our study contributes to this effort.

The *MAOA* ROI characterized in the current study was selected based on prior *in-vitro* analyses, showing that methylation of this region was associated with drastically reduced reporter gene expression (Checknita et al. 2015), and that methylation levels of the ROI were associated with MAO enzymatic activity in the brain (Shumay et al. 2012), highlighting the functional relevance of the *MAOA* ROI. Furthermore, genome-wide methylation studies have pointed to exonic/intronic junction regions, similar to the *MAOA* ROI, as being epigenetic “hot-spots” which remain responsive to epigenetic alterations across the lifespan and are associated with health outcomes (Ashapkin et al. 2017; Ciccarone et al. 2018). As such, our findings showing few age-associated methylation differences further support the *MAOA* ROI as an important target for epigenetic studies, since it may remain responsive to environmental factors as individuals’ age.

The *MAOA* gene is among the most well-established candidate genes associated with behavioural phenotypes (Comai et al. 2012; Byrd and Manuck 2014; Booij et al. 2015; Nilsson et al. 2018). Converging lines of evidence from gene-by-environment studies and from DNA methylation studies have suggested that *MAOA* plays a role in antisocial behaviour, substance misuse, depression (Verhoeven et al. 2012; Melas et al. 2013; Byrd and Manuck 2014; Booij et al. 2015; Melas and Forsell 2015; Checknita et al. 2015, 2020; Bendre et al. 2018; Nilsson et al. 2018), and other mental disorders (Reif et al. 2014; Booij et al. 2015; Ziegler et al. 2016, 2018; Checknita et al. 2018). Among women, adjustments for depression disorders revealed a positive association of depression, but not age, with exonic methylation levels. This is an expected finding given previously reported associations of higher exonic methylation and depression diagnoses in a smaller group of the women included in the current study (Checknita et al. 2018), and further highlights the role of *MAOA* in depression. As such, the comprehensive characterization of *MAOA* ROI methylation, taking account of *MAOA* genotypes, that is reported in current study contributes to furthering understanding of *MAOA* regulation in behavioural phenotypes. It is notable that although our sample was recruited through a substance misuse clinic, almost all the current results were robust to adjustment for substance dependence, depression disorders, anxiety disorders, impulsivity, and tobacco use. This finding suggests that the principal findings may be generalizable to community samples.

## Conclusion

Epigenome studies have shown higher levels of methylation among women than men and, decreasing levels with increasing age in both sexes. The present study observed that within the intronic and exonic regions of *MAOA*, women did present higher levels of methylation but levels did not differ by age, and age did not modify the associations of methylation with sex. This finding is likely attributable to patterns of methylation indicating inactivation of one of the X-chromosomes carried by women. Participants’ ages spanned 3 decades following puberty, and with only a few exceptions, age was not associated with the higher methylation levels among women as compared to men. Older women did display lower levels of CpG13 methylation than younger women, but this finding was not robust to adjustment for MAOA-uVNTR genotype. The stability of methylation in this region across ages needs replication, especially in light of our previous findings, showing that methylation levels in these regions strengthened associations of the interactions of sex-specific genotypes and adversity with aggressive behavior and substance misuse. Few of the observed associations were modified by genotype. None were modified by interactions of genotype and sexual abuse, but sexual abuse of women was associated with slightly increased methylation levels. However, the differences in methylation levels between women who had, and who had not, experienced sexual abuse were small in comparison to the differences in levels between women and men.

## Supplementary Information

Below is the link to the electronic supplementary material.

Supplementary file1 (DOCX 44 kb)

## Data Availability

Data will be made available on reasonable request.
