# Supplementary material for: Associations of age, sex, sexual abuse, and genotype with monoamine oxidase a gene methylation
Source: J Neural Transm (Vienna). 2021 Aug 23;128(11):1721–39. doi: 10.1007/s00702-021-02403-2 (PMC8536631; doi:10.1007/s00702-021-02403-2)
Supplement: Supplementary file 1 — Supplementary file1 (DOCX 44 kb) [file 702_2021_2403_MOESM1_ESM.docx]

Online Resource

**Associations of Age, Sex, Sexual Abuse, and Genotype with Monoamine Oxidase A Gene Methylation**

David Checknita, Jari Tiihonen, Sheilagh Hodgins, Kent W. Nilsson

**Method**

**Processing of samples**. Saliva samples were collected during the meeting between the participant and the researchers, using the Oragene self-collection kit (DNA Genotek®, Canada: www.dnagenotek.com). The samples were stored at room temperature in accordance with manufacturer´s guidelines. DNA was extracted from 200µl of saliva using the silica-based Kleargene DNA extraction method (LGC®, UK: www.lgcgroup.com) and stored at -20^o^C, and subsequently -80^o^C, prior to methylation analysis of the *MAOA* ROI. Genotyping and methylation analyses were performed in a blinded manner.

**Supplementary Tables**

| **Table S1.** Mean methylation levels (%) across the *MAOA* ROI in men and women. | | |
| --- | --- | --- |
| Site | Men | Women |
| CpG1**^a^** | - | - |
| CpG2/3 | 4.6 | 46.7 |
| CpG4 | 16.3 | 43.3 |
| CpG5/6 | 8.8 | 42.1 |
| CpG7/8 | 6.6 | 43.4 |
| CpG9**^a^** | - | - |
| CpG10 | 7.2 | 43.6 |
| CpG11 | 8.5 | 54.6 |
| CpG12 | 16.4 | 31.4 |
| CpG13 | 67.3 | 81.7 |
| CpG14 | 31.2 | 48.4 |
| CpG15 | 77.3 | 73.4 |
| CpG16 | 72.3 | 65.3 |
| Total ROI | 28.4 | 52.3 |
| Exonic CpGs**^b^** | 8.7 | 43.8 |
| Intronic CpGs**^c^** | 44.9 | 59.2 |
| Component 1 CpGs^d^ | 10.3 | 44.6 |
| Component 2 CpGs^e^ | 62.4 | 66.2 |

**^a^**Data not available.

**^b^**Mean methylation level for CpGs 2-10.

**^c^**Mean methylation level for CpGs 11-16.

^d^Mean methylation level for CpGs 2-14.

^e^Mean methylation level for CpGs 15 and 16.

| **Table S2.** Mean methylation (SD) levels (%) across the MAOA ROI in men and women by MAOA uVNTR genotype | | | | | | |
| --- | --- | --- | --- | --- | --- | --- |
|  | **Men** | |  | **Women** | |  |
|  | **MAOA Genotypes** | |  | **MAOA Genotypes** | |  |
| Site | S | L | Statistics | SS | SL / LL | Statistics |
| CpG1**^a^** | - | - |  | - | - |  |
| CpG2/3 | 4.3  (2.752) | 4.9  (1.884) | n.s* | 49.2  (7.061) | 47.7  (9.096) | n.s |
| CpG4 | 15.7  (4.432) | 18.0  (8.844) | n.s | 44.1  (5.790) | 44.4  (7.770) | n.s |
| CpG5/6 | 8.9  (3.343) | 10.6  (9.924) | n.s | 43.8  (5.907) | 43.2  (7.537) | n.s |
| CpG7/8 | 6.8  (3.160) | 6.6  (2.701) | n.s | 44.5  (7.516) | 44.1  (7.809) | n.s |
| CpG9**^a^** | - | - |  | - | - |  |
| CpG10 | 6.7  (3.624) | 8.4  (8.868) | n.s | 45.8  (7.130) | 44.6  (8.410) | n.s |
| CpG11 | 8.7  (4.491) | 8.4  (3.742) | n.s | 56.8  (5.671) | 55.3  (8.588) | n.s |
| CpG12 | 16.8  (4.732) | 16.8  (4.766) | n.s | 31.8  (4.734) | 32.5  (5.834) | n.s |
| CpG13 | 66.7  (9.133) | 67.8  (12.785) | n.s | 81.3  (6.693) | 79.6  (9.529) | n.s |
| CpG14 | 29.9  (7.209) | 32.9  (8.496) | p=.024 | 48.0  (8.198) | 47.9  (8.791) | n.s |
| CpG15 | 79.9  (12.734) | 77.9  (11.014) | n.s | 73.2  (8.821) | 72.8  (10.338) | n.s |
| CpG16 | 73.2  (10.512) | 72.1  (9.559) | n.s | 66.6  (6.523) | 64.7  (8.869) | n.s |
| Total ROI | 28.5  (4.514) | 29.2  (5.141) | n.s | 53.4  (3.876) | 52.7  (6.196) | n.s |
| Exonic**^b^** | 8.5  (2.875) | 9.7  (5.487) | n.s | 45.5  (5.760) | 44.8  (7.322) | n.s |
| Intronic**^c^** | 45.4  (6.699) | 45.5  (7.284) | n.s | 59.59  (4.081) | 58.93  (5.901) | n.s |
| Component 1^d^ | 9.7  2.856 | 10.5  4.193 | n.s | 45.1  5.122 | 44.5  6.810 | n.s |
| Component 2^e^ | 62.4  8.510 | 62.4  10.840 | n.s | 67.0  5.953 | 66.3  7.077 | n.s |

**^a^**Data not available.

**^b^**Mean methylation level for CpGs 2-10.

**^c^**Mean methylation level for CpGs 11-16.

^d^Mean methylation level for CpGs 2-14 in the Full Sample, 2-12 in Men, and 2-12 in Women

^e^Mean methylation level for CpGs 15 and 16 in the Full Sample, 13-16 in Men, and 13-16 in Women

*Not significant at the p<.05 level

| **Table S3.** Summary of models assessing the interaction of sex and age on CpG methylation levels | | | |
| --- | --- | --- | --- |
| Terms | B | p | Bootstrapped 95%  Confidence Interval |
| **CpG2/3** |  |  |  |
| Age | -0.000 | .434 | -0.42 – -0.30 |
| Sex | 0.409 | <.001 | 0.37 – 0.45 |
| Age x Sex | 0.000 | .227 | -0.00 – 0.00 |
| **CpG4** |  |  |  |
| Age | 0.001 | .205 | -0.00 – 0.00 |
| Sex | 0.287 | <.001 | 0.25 – 0.33 |
| Age x Sex | -0.000 | .459 | -0.00 – 0.00 |
| **CpG5/6** |  |  |  |
| Age | 0.001 | .105 | -0.00 – 0.00 |
| Sex | 0.351 | <.001 | 0.31 – 0.39 |
| Age x Sex | -0.00 | .354 | -0.00 – 0.00 |
| **CpG7/8** |  |  |  |
| Age | -0.000 | .817 | -0.00 – 0.00 |
| Sex | 0.368 | <.001 | 0.33 – 0.40 |
| Age x Sex | -0.000 | .853 | -0.00 – 0.00 |
| **CpG10** |  |  |  |
| Age | -0.000 | .448 | -0.00 – 0.00 |
| Sex | 0.379 | <.001 | 0.33 – 0.42 |
| Age x Sex | -0.000 | .665 | -0.00 – 0.00 |
| **CpG11** |  |  |  |
| Age | -0.000 | .837 | -0.00 – 0.00 |
| Sex | 0.461 | <.001 | 0.42 – 0.50 |
| Age x Sex | -0.000 | .755 | -0.00 – 0.00 |
| **CpG12** |  |  |  |
| Age | -0.000 | .543 | -0.00 – 0.00 |
| Sex | 0.158 | <.001 | 0.13 – 0.19 |
| Age x Sex | -0.000 | .808 | -0.00 – 0.00 |
| **CpG13** |  |  |  |
| Age | -0.000 | .160 | -0.00 – 0.00 |
| Sex | 0.182 | <.001 | 0.12 – 0.24 |
| Age x Sex | -0.002 | .019* | -0.00 – 0.00 |
| **CpG14** |  |  |  |
| Age | -0.000 | .732 | -0.00 – 0.00 |
| Sex | 0.165 | <.001 | 0.12 – 0.21 |
| Age x Sex | -0.000 | .902 | -0.00 – 0.00 |
| **CpG15** |  |  |  |
| Age | 0.003 | .009 | -0.00 – 0.00 |
| Sex | 0.005 | .854 | -0.05 – 0.06 |
| Age x Sex | -0.002 | .025 | -0.00 – -0.00 |
| **CpG16** |  |  |  |
| Age | -0.000 | .513 | -0.00 – 0.00 |
| Sex | -0.064 | .011 | -0.11 - -0.01 |
| Age x Sex | -0.000 | .616 | -0.00 – 0.00 |

^a^ Mediation analysis conducted using bootstrapped bias-corrected 95% confidence

intervals with 5000 bootstrapping samples.

*Subsequent conditional effects analysis of this interaction term showed significant

interactions among all 3 age groups of women in the same direction, thus indicating

a sex difference rather than a true interaction.

| **Table S4**. Comparison of exonic, intronic, and component methylation levels by experience of sexual abuse among women | | | |
| --- | --- | --- | --- |
| **Methylation** | **Women with No SA**  **(n=82)** | **Women with SA**  **(n=62)** | **Statistics** |
| Mean (SD) CpG 2-10 Methylation (Exon 1) | 43.12  (5.883) | 45.95  (5.533) | F(1,143)0.63 p=.004 |
| Mean (SD) CpG 11-16 Methylation (Intron 1) | 59.52  (3.439) | 58.91  (4.014) | F(1,142)0.69 p=.332 |
| Mean (SD) CpG 2-14 Methylation (component 1) | 47.87  (4.922) | 49.84  (4.178) | F(1,142)2.35 p=.012 |
| Mean (SD) CpG 15 &16 Methylation (component 2) | 69.89  (5.485) | 67.77  (6.700) | F(1,143)1.27 p=.043 |

| **Table S5**. Comparison of exonic, intronic, and component methylation levels by experience of sexual abuse among men | | | |
| --- | --- | --- | --- |
| **Methylation** | **Men with No SA**  **(n=75)** | **Men with SA**  **(n=13)** | **Statistics** |
| Mean (SD) CpG 2-10 Methylation (Exon 1) | 8.70  (1.658) | 8.29  (2.198) | F(1,87)1.65 p=.443 |
| Mean (SD) CpG 11-16 Methylation (Intron 1) | 45.50  (7.717) | 43.45  (6.182) | F(1,87)0.51 p=.367 |
| Mean (SD) CpG 2-14 Methylation (component 1) | 18.80  (3.060) | 17.53  (2.910) | F(1,87)0.04 p=.168 |
| Mean (SD) CpG 15 &16 Methylation (component 2) | 75.18  (10.66) | 72.23  (11.488) | F(1,87)0.07 p=.366 |

**Table S6.** General Linear Models of factors associated with *MAOA* methylation in exonic and intronic regions and components 1 and 2 with siblings removed from analyses

|  | **Methylation** | | | | | | | | | | | | | | | |
| --- | --- | --- | --- | --- | --- | --- | --- | --- | --- | --- | --- | --- | --- | --- | --- | --- |
|  | **Exonic** | | |  | **Intronic** | | |  | **Component 1** | | |  | **Component 2** | | | |
|  | df | F | p |  | df | F | p |  | df | F | p |  | df | F | p |  |
| MAOA-uVNTR | 1 | 1.742 | .187 |  | 1 | 0.003 | .958 |  | 1 | 3.129 | .077 |  | 1 | 0.094 | .759 |  |
| Sexual abuse | 1 | 3.271 | .071 |  | 1 | 0.000 | .991 |  | 1 | 1.651 | .199 |  | 1 | 0.075 | .785 |  |
| Sex | 1 | 1148.255 | <.001 |  | 1 | 142.200 | <.001 |  | 1 | 795.476 | <.001 |  | 1 | 0.264 | .608 |  |
| Sexual abuse x Sex |  | 9.445 | .002 |  |  | 0.008 | .928 |  |  | 3.397 | .065 |  |  | 0.202 | .653 |  |
| MAOA-uVNTR x Sexual Abuse |  | 0.401 | .526 |  |  | 1.154 | .283 |  |  | 1.018 | .313 |  |  | 0.383 | .536 |  |
| MAOA-uVNTR x Sex |  | 3.823 | .051 |  |  | 0.165 | .685 |  |  | 5.190 | .023 |  |  | 0.164 | .686 |  |
| MAOA-uVNTR x Sexual Abuse x Sex |  | 0.236 | .627 |  |  | 1.203 | .273 |  |  | 0.863 | .353 |  |  | 0.958 | .328 |  |
